# Steroid receptor coactivators, HER-2 and HER-3 expression is stimulated by tamoxifen treatment in DMBA-induced breast cancer

**DOI:** 10.1186/1471-2407-12-247

**Published:** 2012-06-15

**Authors:** Line L Haugan Moi, Marianne Hauglid Flågeng, Jennifer Gjerde, Andre Madsen, Therese Halvorsen Røst, Oddrun Anita Gudbrandsen, Ernst A Lien, Gunnar Mellgren

**Affiliations:** 1 Institute of Medicine, University of Bergen, Bergen, N-5021, Norway; 2 Hormone Laboratory, Haukeland University Hospital, N-5021, Bergen, Norway; 3 Department of Clinical Pathology, University Hospital of North Norway, N-9038, Tromsø, Norway

**Keywords:** SRC-1, SRC-2/TIF-2, SRC-3/AIB1, HER, HER-2, Breast cancer, Tamoxifen

## Abstract

**Background:**

Steroid receptor coactivators (SRCs) may modulate estrogen receptor (ER) activity and the response to endocrine treatment in breast cancer, in part through interaction with growth factor receptor signaling pathways. In the present study the effects of tamoxifen treatment on the expression of SRCs and human epidermal growth factor receptors (HERs) were examined in an animal model of ER positive breast cancer.

**Methods:**

Sprague-Dawley rats with DMBA-induced breast cancer were randomized to 14 days of oral tamoxifen 40 mg/kg bodyweight/day or vehicle only (controls). Tumors were measured throughout the study period. Blood samples and tumor tissue were collected at sacrifice and tamoxifen and its main metabolites were quantified using LC-MS/MS. The gene expression in tumor of SRC-1, SRC-2/transcription intermediary factor-2 (TIF-2), SRC-3/amplified in breast cancer 1 (AIB1), ER, HER-1, -2, -3 and HER-4, as well as the transcription factor Ets-2, was measured by real-time RT-PCR. Protein levels were further assessed by Western blotting.

**Results:**

Tamoxifen and its main metabolites were detected at high concentrations in serum and accumulated in tumor tissue in up to tenfolds the concentration in serum. Mean tumor volume/rat decreased in the tamoxifen treated group, but continued to increase in controls. The mRNA expression levels of SRC-1 (*P* = 0.035), SRC-2/TIF-2 (*P* = 0.002), HER-2 (*P* = 0.035) and HER-3 (*P* = 0.006) were significantly higher in tamoxifen treated tumors compared to controls, and the results were confirmed at the protein level using Western blotting. SRC-3/AIB1 protein was also higher in tamoxifen treated tumors. SRC-1 and SRC-2/TIF-2 mRNA levels were positively correlated with each other and with HER-2 (*P* ≤ 0.001), and the HER-2 mRNA expression correlated with the levels of the other three HER family members (*P* < 0.05). Furthermore, SRC-3/AIB1 and HER-4 were positively correlated with each other and Ets-2 (*P* < 0.001).

**Conclusions:**

The expression of SRCs and HER-2 and -3 is stimulated by tamoxifen treatment in DMBA-induced breast cancer. Stimulation and positive correlation of coactivators and HERs may represent an early response to endocrine treatment. The role of SRCs and HER-2 and -3 should be further studied in order to evaluate their effects on response to long-term tamoxifen treatment.

## Background

Breast cancer is the most frequent malignancy and a major cause of cancer deaths in women. It is well established that estrogen has pro-carcinogenic effects in mammary epithelium by stimulating proliferation and leaving the cells prone to mutations during cell cycle progression [1]. The selective estrogen receptor modulator (SERM) tamoxifen is widely used in ER positive breast cancer where it improves disease-free and overall survival [2]. Tamoxifen would normally function as an ER antagonist in breast cancer by binding to the ER and inducing conformational changes which favor corepressor recruitment and inhibit ER mediated gene transcription. However, tamoxifen demonstrates ER agonistic effects in other tissues such as bone and liver. The expression and activity of nuclear receptor coactivators have been pointed out as the main determinants of tissue- and cell specific effects of tamoxifen [3].

The SRC family includes SRC-1, SRC-2/TIF-2 and SRC-3/AIB1. The SRCs have similar structural and functional properties, but are genetically distinct, exhibit tissue-specific differences in expression profiles and are suggested to be involved in various diseases, including human cancers [4]. All three SRCs are expressed in normal and malignant breast tissue [5,6]. SRC-3/AIB1 is now considered to be an oncogene [7], which is overexpressed in more than 30% and genetically amplified in 5 – 10% of breast tumors [8-11]. In cellular assays, overexpression of SRC-3/AIB1 has been associated with a shift toward ER agonistic effects of tamoxifen and growth of malignant cells during endocrine treatment [12], whereas dissociation of SRC-3/AIB1 from ER has been shown to restore sensitivity in tamoxifen resistant cells [13]. SRC-1 has also been shown to contribute to the agonistic properties of 4-hydroxytamoxifen (4OHtam) [14]. At the clinical level, overexpression of SRC-1 or SRC-3/AIB1 has been associated with resistance to endocrine treatment and reduced disease-free survival, especially when overexpressed together with HER-2, also known as HER-2/neu or erbB2 [15-17]. HER-2 signaling is targeted in breast cancer therapy using specific antibodies such as trastuzumab or tyrosine kinase inhibitors. Studies of coactivators and HER-2 levels in breast tumor tissue during endocrine treatment may reveal important regulatory mechanisms of relevance to endocrine sensitivity, treatment response and patient outcome over time.

We have previously reported that 4 weeks of preoperative treatment with tamoxifen in the 1-20 mg dose range led to significant upregulation of SRC-1, SRC-2/TIF-2 and SRC-3/AIB1 mRNA in human breast cancer tissue [6]. SRC-3/AIB1 and HER-2 mRNA levels did correlate, and higher SRC-3/AIB1 mRNA levels in tumor at surgery were associated with reduced disease-free survival after a median follow-up time of 8 years. During estrogen deprivation using aromatase inhibitors we found SRC-1 and HER-2 mRNA to be upregulated [18]. Interestingly, this upregulation was particularly evident among therapy responders, again underlining a potential relationship between endocrine treatment, SRCs, HER-2 and treatment response that should be further explored.

In the present study we used an animal model of hormone dependent breast cancer induced by 7,12-dimethylbenz(a)anthracene (DMBA) [19] to study the effect of tamoxifen therapy on expression levels of SRC-1, SRC-2/TIF-2, SRC-3/AIB1 and HER-2 in tumor tissue. We also analyzed the mRNA expression of HER-1 (also known as epidermal growth factor receptor EGFR), HER-3 and HER-4, known to share functional properties with HER-2 [20], but much less studied in breast cancer. We also analyzed the expression of the transcription factor Ets-2, that is known to interact with the SRCs, and ERα. We found tamoxifen and its main metabolites at high concentrations in serum and accumulated in tumor tissue with a clear treatment response in the tamoxifen treated tumors. The mRNA and protein expression levels of SRCs, HER-2 and HER-3 were significantly higher in tamoxifen treated tumors compared to controls. Interestingly, SRC-1 and SRC-2/TIF-2 mRNA levels were correlated with each other and with HER-2. SRC-3/AIB1 and HER-4 were positively correlated with each other and with Ets-2.

## Methods

### Animal model

Non-immunized female SPF Sprague-Dawley rats of stock NTac:SD from Taconic M&B (Borup, Denmark) were administered a single dose of 20 mg DMBA (D-3254; Sigma-Aldrich Norway AS, Oslo, Norway) at age three weeks. After ten weeks all rats had developed palpable tumors, and a total of 16 Sprague-Dawley rats were randomized into two different experimental groups according to treatment. The tamoxifen group received tamoxifen dissolved in peanut oil once daily by gastric tube at a dose of 40 mg/kg bodyweight whereas control rats were administered vehicle only (peanut oil) in corresponding amounts (2.8 ml/kg body weight). The rats were weighed every third day for calculations of treatment dosage, and treated for 13 days before being sacrificed on day 14. A longer treatment period would result in a higher proportion of deaths among the controls and was not considered ethically acceptable. Tumors were counted and measured by calliper throughout the study period, and tumor volumes calculated using the formula: (length) x (width^2^)/2. The relative tumor volumes were calculated as the ratio of the tumor volume on day *n* divided by the tumor volume on day 0.

**Figure 1:**
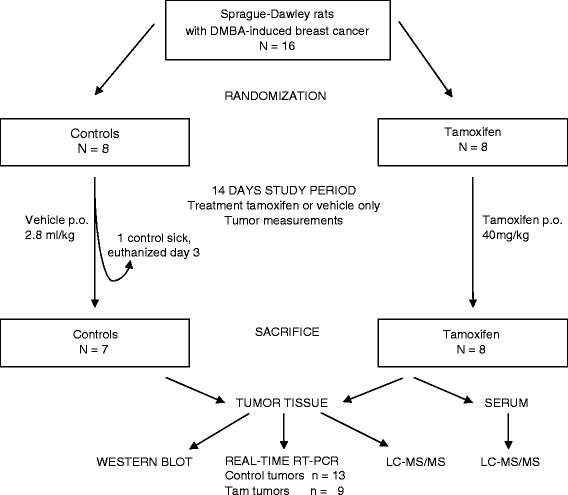
**Schematic presentation of the study model.** 16 Sprague-Dawley rats with DMBA-induced endocrine sensitive breast cancer were randomized to treatment with oral tamoxifen or vehicle only for 14 days. The rats were weighed every third day for calculation of treatment dosage and the tumors were counted and measured. Blood was sampled at the end of the study for measurements of tamoxifen and metabolites, and tumor tissue was collected for gene expression measurements of SRCs and the growth factor receptors HER-1 to -4. Tumor tissue was also used for protein analyses of SRCs and HERs by Western blotting and for drug measurements using LC-MS/MS.

On day 14, the rats were anaesthetized with 2 – 5% isoflurane (Forene: Abbott Scandinavia AB, Solne, Sweden) mixed with oxygen and nitrous oxide. Blood was collected from the heart in BD Vacutainer tubes with no additive (Becton Dickinson and Co., Plymouth, UK). Tumor tissue was collected immediately post mortem and freeze-clamped before storage at **-**80 °C until further analyses. The study model is presented in Figure 1.

The rats received a standard diet from B & K Universal (Nittedal, Norway), had free access to tap water and feed, and were kept in a room with 12 h light/dark cycles and a constant temperature of 20 °C ± 3 °C throughout the experiment. The study was approved by the Norwegian State Board of Biological Experiments with Living Animals.

### RNA extraction, reverse transcription and real-time PCR

Tumor tissue was homogenized manually using mini-pestils and RNA extracted using Trizol (Invitrogen, Carlsbad, CA, USA) according to the manufacturer’s instructions. The quality and quantity of total RNA in each sample was analyzed using the NanoDrop (Saveen Werner, Copenhagen, Denmark) and 1 μg total RNA used for reverse transcription with the Transcriptor First Strand cDNA Synthesis kit (Roche, Mannheim, Germany).

Real-time PCR-reactions were performed according to the protocol on a LightCycler 480 instrument (Roche) using gene specific primers (Biomers.net, Ulm, Germany), Universal ProbeLibrary probes and the kit LightCycler 480 Probes Master (Roche). The primer sequences and probe numbers were as follows: SRC-1 tgctcccgaggaggttaaa (s) and atcaaactggtcaaggtcagc (as), probe #21; SRC-2/TIF-2 ctgtgaaggaggaggtgagc (s) and tccaaaatctcttccaagttgtc (as), probe #64; SRC-3/AIB1 ctggtgctgctgtgatgag (s) and gccatttgggcattaaagaa (as), probe #3; HER-2 tgtggatctggatgaacgag (s) and cactacagttgcaatgatgaatgt (as), probe #3; HER-1 cagagctgaaaaggactgcaa (s) and cacattctggcaggagacac (as), probe #3; HER-3 caacccccataccaagtatca (s) and acgtctggtccaccacaaa (as), probe #25; HER-4 caataggagtgaaattggacaca (s) and ccatcctggtacacaaactgac (as), probe #63; ERα tttctttaagagaagcattcaagga (s) and ttatcgatggtgcattggttt (as), probe #130; Ets-2 gccctacgccttcgtctc (s) and ttgattccaaaatcattcatcg (as), probe #70; TATA-box binding protein (TBP) cccaccagcagttcagtagc (s) and caattctgggtttgatcattctg (as), probe #129.

Quantification was performed using external standard curves for each target gene with serially diluted cDNA from a cDNA-stock made by pooling all study samples. mRNA expression levels were calculated relative to that of the housekeeping gene TBP.

### Protein extraction and western blot analysis

Protein was extracted from tumor tissue after homogenization of tissue twice at 25 Hz for 2 minutes using a TissueLyser (Qiagen, Düsseldorf, Germany) in RIPA lysis buffer (Thermo Scientific, Belgium) containing 2 mM EDTA, 0.5 mM phenylmethylsulfonyl fluoride (PMSF, Sigma Aldrich, St. Louis, MO) and protease inhibitors (Complete mini-EDTA free protease inhibitor cocktail tablet, Roche). Lysates were incubated on ice for 10 minutes prior to centrifugation at 12.000 x g for 20 min at 4 °C, and the supernatant was collected and stored at -80 °C. Protein concentrations were determined by the Lowry method using RC DC Protein Assay (BioRad, Hercules, CA, USA). 145 ug total protein per sample was resolved on 4-20% TXD Mini protean RGX precast gels (Biorad) and transferred to nitrocellulose membranes using the Trans-Blot Turbo transfer system (Biorad) for 9 minutes at 2.5 A constant up to 25 V. Membranes were incubated for 1 h at room temperature in blocking solution containing 5% skimmed milk in Phosphate-buffered saline with Tween-20 (PBS-T), followed by rinsing in PBS-T before incubation for 1 h in room temperature with specific primary antibodies for HER-2 (anti-erbB-2, Millipore, Billerica, MA, USA; 1:500), HER-3 (ErbB-3, Santa Cruz; 1:200), SRC-1 (BD Bioscience, San Joes, CA, USA; 1:500), SRC-2/TIF-2 (BD Bioscience; 1:500) and SRC-3/AIB1 (Cell Signaling, Boston, MA, USA; 1:500). Membranes were rinsed in PBS-T before incubation for 40 minutes with either goat-anti-mouse secondary antibody (BD Bioscience, 1:5000) or goat-anti-rabbit secondary antibody (Thermo Science, 1:10000). Membranes were washed in PBS-T and proteins were detected by SuperSignal West Femto (Thermo Scientific, Rockford, IL, US) using a ChemiDoc System (BioRad). Membranes were stripped using Restore Western Blot Stripping buffer (Thermo Scientific) for 45 minutes, washed in PBS-T for detection of reference protein using primary antibody to β-actin (Abcam, Cambridge, UK; 1:5000) and secondary antibody donkey-anti-mouse (Santa Cruz, 1:5000) following the protocol above.

### Tamoxifen and metabolite concentrations

Tamoxifen and its metabolites 4OHtam, *N*-desmethyltamoxifen (NDtam), *N*-desdimethyltamoxifen (NDDtam), tamoxifen-*N*-oxide (tamNox) and 4-hydroxy-*N*-desmethyltamoxifen (4OHNDtam) were measured in serum by high-pressure liquid chromatography-tandem mass spectrometry (LC-MS/MS) as previously published [21]. Before measuring tamoxifen and metabolites in tumor, about 0.4 g tissue was homogenized in ice-cold 50 mM Tris-HCl buffer (1:5, (w/v)) with pH 7.4 at 26,000 rev/min. The homogenates were mixed with an equal volume of 100% acetonitrile and the precipitated proteins were removed by centrifugation at 15.000 × g for 20 min prior to LC-MS/MS analyses [22]. Using this procedure, we have earlier observed a recovery for tamoxifen, 4OHtam, NDtam, NDDtam and 4OHNDtam in the range 69-110% in seven different rat tissues [23].

### Statistics

Since the mRNA expression levels are not normally distributed, differences between the treatment groups were analyzed using non-parametric Mann-Whitney *U* test. Any correlation between expression levels of the different target genes, between target genes mRNA and tumor volume measurements and correlations between tamoxifen metabolites were investigated using Spearman’s correlation. The level of statistical significance was set at *P* < 0.05. The SPSS software package version 18.0 (SPSS, Chicago, USA) was used for all statistical analyses.

## Results

### Animal weights, tumor measurements and treatment response to tamoxifen

**Figure 2:**
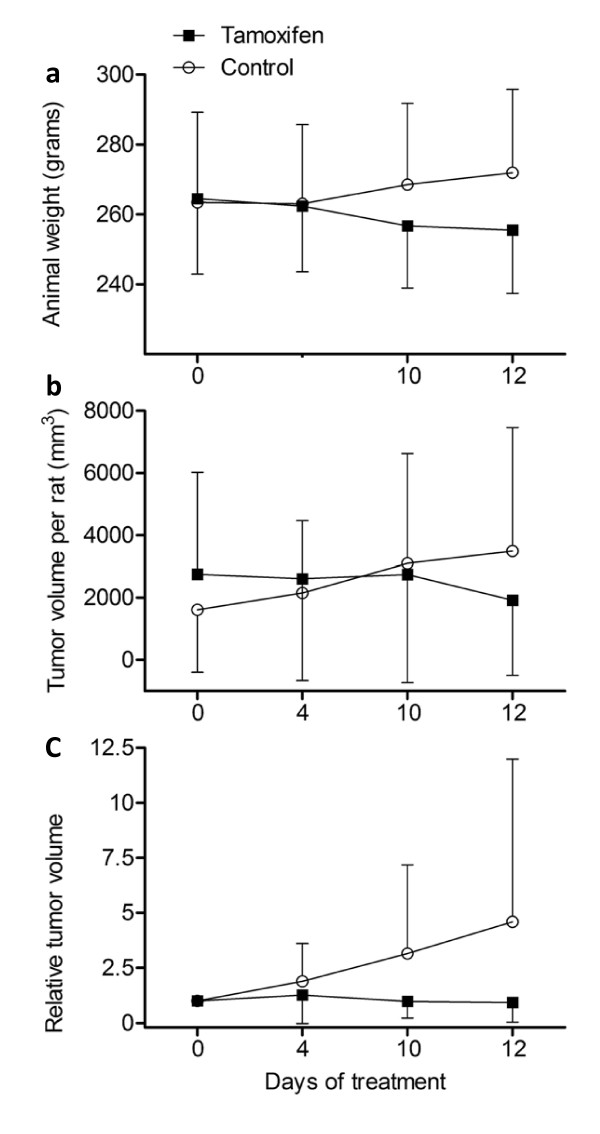
**Animal weight and tumor volume during tamoxifen treatment.** Rats with DMBA-induced ER positive breast cancers were orally treated with either tamoxifen at a dose of 40 mg/kg bodyweight/day or received vehicle only for 14 days. Animals were weighed every third day for calculation of treatment dosage. Mean weights (± SD) are presented in the graph (A). Tumor number and size were measured every second day and the tumor volumes were calculated according to the formula (LxW^2^)/2. The mean tumor volume/rat (B) and mean relative tumor volume/rat compared to day 0 (C) during the treatment period are presented according to treatment group.

The animals in the control group increased in weight from a mean (± SD) of 263 g (± 21) on day 0 to 272 g (± 24) on day 12. In the tamoxifen treated animals, the mean weight fell from 265 g (± 25) to 256 g (± 18) (Figure 2A). Correspondingly, mean tumor volume during tamoxifen treatment dropped from 2750 to 1923 mm^3^/rat (Figure 2B), and the mean relative tumor volume on day 13 was 0.9 in tamoxifen treated rats (Figure 2C). In contrast, the controls experienced an increase in average tumor volume in the same time period, from 1611 to 3488 mm^3^/rat, and the mean relative tumor volume was 4.6 on day 13 (Figure 2B and C). The variation in mean tumor volume per rat was considerable in both tamoxifen treated and control rats (Figure 2B). It should also be noted that one of the rats in the control group had to be euthanized on day 3 of treatment due to severe illness and was excluded from the study after study start.

At the start of the treatment period, the tumors were equally distributed between the treatment groups with an average number of 2.4 tumors/rat (± 1.8) in the group which received tamoxifen treatment compared to 2.5 tumors/rat (± 2.3) in the control group. Of the 19 tumors in the tamoxifen treated rats, one tumor disappeared, 13 tumors demonstrated regression whereas five tumors increased in size. Four out of the 20 tumors in control rats demonstrated a reduction in size, whereas the remaining 16 tumors increased in size and additional eight tumors appeared during the study period. We observed new tumors during tamoxifen treatment, but the mean number of tumors per rat leveled out and reached 3.0 (± 3.6) during the treatment period whereas the control animals experienced a continuous increase also in tumor number to 4.0 (± 1.9) at the end of the study. However, we observed that growing tumors could confluence, whereas tumors in regression could disintegrate into several smaller tumors, making the number of tumors a poor marker of treatment response.

### mRNA expression of SRCs, HER growth factor receptors, ERα and Ets-2

**Figure 3:**
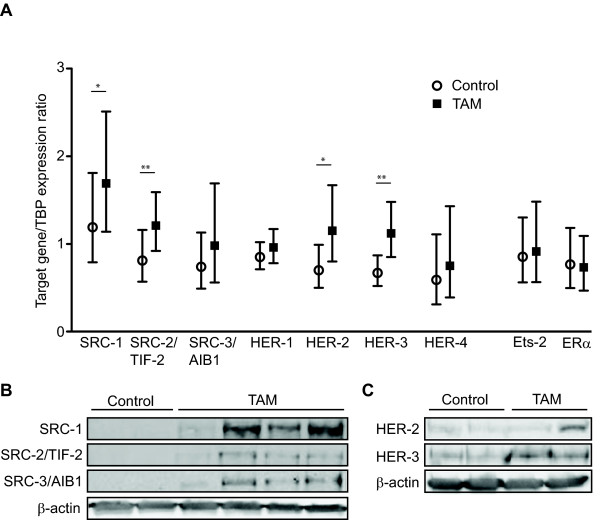
**SRCs and HERs expression during tamoxifen treatment.** SRC-1, -2 and -3 and HER-1, -2, -3 and -4 mRNA expression levels as well as the levels of Ets-2 and ERα mRNA after 14 days of oral tamoxifen treatment are presented compared to controls receiving vehicle only in DMBA-induced breast cancer. The mRNA levels of our target genes were calculated relative to the expression of the housekeeping gene TBP and the data presented as geometric means with error bars indicating 95% confidence intervals. Differences in mRNA levels between the treatment groups were evaluated using Mann Whitney *U* test and statistical significance indicated in the figure (* = *P* < 0.05, ** = *P* < 0.01) (A). The protein levels of the SRCs (B) and HER-2 and HER-3 (C) in tamoxifen treated tumors compared to controls were analyzed using Western blots. Representative blots of the tumor response to tamoxifen treatment are presented, using β-actin as control for protein load.

Tumors too small for RNA extraction according to protocol had to be excluded from further analyses. Thus, 13 representative tumors from the seven remaining control animals were analyzed for mRNA expression. For one of the tamoxifen treated animals, no tumors were observed at the end of the study and for an additional two animals the remaining tumor was too small for RNA extraction, leaving a representative selection of nine tumors from five tamoxifen treated animals for gene expression analyses. Gene expression analysis by real-time RT-PCR demonstrated a significant upregulation of SRC-1 during tamoxifen treatment. The geometric mean (with 95% confidence interval) of the SRC-1 mRNA levels relative to the housekeeping gene TBP in tamoxifen treated tumors was 1.69 (1.14 – 2.51) compared to control animals 1.19 (0.79 – 1.81) (*P* = 0.035, Figure 3A). SRC-2/TIF-2 was also significantly higher in tamoxifen treated tumors with mRNA levels of 1.21 (0.92 – 1.59) compared to 0.81 (0.57 – 1.16) in control tumors (*P* = 0.002). The geometric mean of SRC-3/AIB1 mRNA levels during tamoxifen treatment was 0.98 (0.56 – 1.69) which was higher, but not significantly different from levels in tumors from control animals. However, the mRNA levels of SRC-3/AIB1 were significantly positively correlated with SRC-2/TIF-2 (*P* = 0.023). SRC-1 and SRC-2/TIF-2 expression levels were highly positively correlated (*P* < 0.001, Table 1).

**Table 1:** Correlations between the mRNA expression of coactivators, HER growth factor receptors, Ets-2 and ERα

	**SRC-1**		**SRC-2/TIF-2**		**SRC-3/AIB1**		**HER-1**		**HER-2**		**HER-3**		**HER-4**		**Ets-2**	
	r	*P*	r	*P*	r	*P*	r	*P*	r	*P*	r	*P*	r	*P*	r	*P*
**SRC-2/TIF-2**	0.741	<0.001**	-	-												
**SRC-3/AIB1**	0.141	0.53	0.483	0.023*	-	-										
**HER-1**	0.260	0.242	0.335	0.128	0.293	0.186	-	-								
**HER-2**	0.659	0.001**	0.714	<0.001**	0.355	0.105	0.477	0.025*	-	-						
**HER-3**	0.395	0.069	0.467	0.028*	0.071	0.755	0.354	0.106	0.579	0.005**	-	-				
**HER-4**	0.475	0.026*	0.596	0.003**	0.717	<0.001**	0.224	0.316	0.482	0.023*	0.280	0.208	-	-		
**Ets-2**	0.313	0.156	0.495	0.019*	0.778	<0.001**	0.214	0.339	0.554	0.007**	0.149	0.510	0.789	<0.001**	-	-
**ERα**	0.320	0.146	0.338	0.124	-0.015	0.946	0.099	0.662	0.389	0.074	0.088	0.699	0.235	0.291	0.264	0.236

Spearman’s nonparametric test. Correlation coefficients (r) with *P*-values are presented in the table. Significant correlations are indicated by italics and * = *P* < 0.05 or ** = *P* < 0.01

We also observed a significant upregulation of HER-2 and HER-3 mRNA levels during endocrine treatment. HER-2 mRNA levels had a geometric mean of 1.15 (0.80 – 1.67) in tamoxifen treated tumors compared to 0.70 (0.50 – 0.99) in controls (*P* = 0.035, Figure 3A) and HER-3 mRNA was 1.12 (0.85 – 1.48) during tamoxifen treatment and 0.67 (0.52 – 0.87) in tumors from controls (*P* = 0.006). HER-2 and HER-3 were also significantly positively correlated (*P* = 0.005, Table 1). There were no significant differences in HER-1 and HER-4 mRNA levels between tamoxifen treated and control tumors (Figure 3A). However, the mRNA levels of HER-2 correlated with HER-1 (*P* = 0.025), HER-3 (*P* = 0.005), HER-4 (*P* = 0.023), and most clearly with SRC-1 and SRC-2/TIF-2 (*P* ≤ 0.001). Although expression of SRC-3/AIB1 and HER-4 did not increase significantly during tamoxifen treatment, the respective mRNA levels were highly positively correlated (*P* < 0.001, Table 1).

The transcription factor Ets-2 mRNA levels were not found to be different in tamoxifen treated tumors compared to controls (Figure 3A). Interestingly, however, Ets-2 was positively correlated with the mRNA expression of SRC-3/AIB1 and HER-4 (Table 1). ERα mRNA expression was lower in tamoxifen treated tumors with a geometric mean of 0.73 (0.48 – 1.11), but not significantly different from the levels in control tumors of 0.77 (0.50 – 1.18) (*P* = 0.65) (Figure 3A).

SRC-2/TIF-2 tended to be higher in the tumors with the largest volume at the end of the study (*P* = 0.059). Overall, we did not find any significant correlation between tumor volume/rat, relative tumor volume and the expression of the individual mRNAs in tumor (Table 2).

**Table 2:** Correlations between the mRNA expression of coactivators, HER growth factor receptors, Ets-2 and ERα and tumor volume

	**Tumor volume**		**Relative tumor volume**	
	r	*P*	r	*P*
**SRC-1**	0.210	0.513	−0.168	0.602
**SRC-2/TIF-2**	0.559	0.059	−0.084	0.795
**SRC-3/AIB1**	0.497	0.101	−0.154	0.633
**HER-1**	−0.070	0.829	−0.322	0.308
**HER-2**	0.301	0.342	−0.273	0.391
**HER-3**	0.280	0.379	−0.203	0.527
**HER-4**	0.350	0.265	−0.063	0.846
**Ets-2**	0.329	0.297	0.112	0.729
**ERα**	0.175	0.587	0.259	0.417

Spearman’s nonparametric test. Correlation coefficients (r).

with *P*-values are presented in the table.

### Protein expression of steroid receptor coactivators, HER-2 and HER-3

The ability of tamoxifen treatment to induce the expression of SRC-1, SRC-2/TIF-2, SRC-3/AIB1, HER-2 and HER-3 in tumor tissue was also determined at the protein level, using Western blotting on protein extracts from tamoxifen treated tumors and controls. SRC-1, SRC-2/TIF-2 and SRC-3/AIB1 proteins were found to be expressed at higher levels in tamoxifen treated tumors compared to controls, as demonstrated in Figure 3B.

Moreover, HER-3 expression was clearly induced by tamoxifen at the protein level confirming the results above at the mRNA level (Figure 3C). Although the Western blots suggested a variable degree of HER-2 expression after tamoxifen treatment, several of the tamoxifen treated tumors also demonstrated higher protein levels of HER-2 compared to untreated controls (Figure 3C).

### Tamoxifen and metabolites in serum and tumor tissue

Tissue from five tamoxifen treated tumors was used for measurements of tamoxifen and its metabolites. Noteworthy, two tumors and two serum samples from control animals were also analyzed for tamoxifen and metabolites as control. Tamoxifen and the five metabolites 4OHtam, NDtam, 4OHNDtam, NDDtam and tamNox were detectable in all serum samples from tamoxifen treated rats, but were not detectable in the negative controls. The median tamoxifen concentration was 203 ng/ml with interquartile range (quartile 1 - quartile 3) of 184 - 229 ng/ml. The pharmacologically active metabolite of tamoxifen, 4OHtam, had a median concentration of 372 (319 - 499) ng/ml, but the dominating metabolite in serum was the other hydroxylated tamoxifen metabolite, 4OHNDtam, with a median concentration of 552 (427 - 593) ng/ml. NDDtam was found to have the lowest level in serum with median concentration of 4.7 (4.4 - 6.5) ng/ml (Table 3).

**Table 3:** Tamoxifen and metabolites in serum and tumor tissue during oral tamoxifen treatment

	**Serum**^#^	Tumor*	**Ratio**^‡^
	**(n = 8)**	**(n = 5)**	**(n = 5)**
	**Median (q1-q3)**	**Median (q1-q3)**	**Median (q1-q3)**
**Tam**	203 (184-229)	11750 (7000-15475)	50 (36-74)
**4OHtam**	372 (319-499)	18850 (8625-25775)	36 (23-72)
**4OHNDtam**	552 (427-593)	48850 (26550-76750)	92 (45-136)
**NDtam**	371 (335-417)	33200 (18225-49825)	93 (46-136)
**NDDtam**	4.7 (4.4-6.5)	376 (152-688)	84 (36-164)
**TamNox**	159 (127-180)	49 (29-685)	0.3 (0.2-3.9)
**4OHtam/tam**^**†**^	1.9 (1.7-2.2)	1.5 (1.2-1.7)	-
**NDtam/tam**^**†**^	1.7 (1.5-2.0)	2.8 (2.5-3.2)	-
**NDDtam/tam**^**†**^	0.02 (0.02-0.04)	0.04 (0.02-0.05)	-

^#^ Tamoxifen and metabolite measurements are presented as ng/ml serum.

*Tamoxifen and metabolite measurements are presented as ng/g tumor tissue.

^‡^ Tumor/serum ratios of concentrations of tamoxifen and metabolites.

^**†**^ Concentration ratios of metabolite/tamoxifen.

Tam = tamoxifen; 4OHtam = 4-hydroxytamoxifen; 4OHNDtam = 4-hydroxy-*N*-desmethyltamoxifen; NDtam = *N*-desmethyltamoxifen; NDDtam = *N*-desdimethyltamoxifen; TamNox = tamoxifen-*N*-oxide; q1-q3 = quartile 1 – quartile 3

Tamoxifen and its hydroxylated and demethylated metabolites accumulated in tumor tissue with median tumor to serum concentration ratios ranging from 36 to 93 (Table 3). As opposed to the other metabolites, both NDDtam and tamNox were detected at lower concentrations than the parent drug in serum samples and tumor tissue.

The serum levels of the demethylated metabolites NDtam and 4OHNDtam were significantly positively correlated in serum (*P* = 0.002, Table 4). With only tumor tissue from five tumors available for metabolite measurements, the results have to be interpreted with caution. However, a significant positive correlation between the concentration of tamoxifen and the main metabolites identified in tumor tissue was observed: 4OHtam, 4OHNDtam and NDtam (*P* <0.001, Table 4). TamNox was the only metabolite whose concentrations in serum and tumor correlated (*P* = 0.04).

**Table 4:** Correlations between tamoxifen metabolite concentrations in tumor tissue and serum.

		**Tam**		**4OHtam**		**4OHNDtam**		**NDtam**		**NDDtam**		**TamNox**	
		Tumor	Serum	Tumor	Serum	Tumor	Serum	Tumor	Serum	Tumor	Serum	Tumor	Serum
**Tam**													
Tumor	r	**-**											
	n	**-**											
Serum	r	0.70	-										
	n	5	-										
**4OHtam**													
Tumor	r	1.00**	0.70	-									
	n	5	5	-									
Serum	r	−0.70	−0.19	−0.70	-								
	n	5	8	5	-								
**4OHNDtam**													
Tumor	r	1.00**	0.70	1.00**	−0.70	-							
	n	5	5	5	5	-							
Serum	r	−0.60	0.41	−0.60	−0.31	−0.60	-						
	n	5	8	5	8	5	-						
**NDtam**													
Tumor	r	1.00**	0.70	1.00**	−0.70	1.00**	−0.60	-					
	n	5	5	5	5	5	5	-					
Serum	r	−0.70	0.14	−0.70	0.00	−0.70	0.91**	−0.70	-				
	n	5	8	5	8	5	8	5	-				
**NDDtam**													
Tumor	r	0.40	0.60	0.40	−0.10	0.40	−0.20	0.40	−0.60	-			
	n	5	5	5	5	5	5	5	5	-			
Serum	r	−0.50	−0.12	−0.50	−0.24	−0.50	−0.52	−0.50	−0.48	0.40	-		
	n	5	8	5	8	5	8	5	8	5	-		
**TamNox**													
Tumor	r	0.80	0.50	0.80	−0.50	0.80	−0.60	0.80	−0.50	−0.10	−0.70	-	
	n	5	5	5	5	5	5	5	5	5	5	-	
Serum	r	0.90*	0.41	0.90*	0.24	0.90*	0.43	0.90*	0.55	0.30	−0.21	0.90*	-
	n	5	8	5	8	5	8	5	8	5	8	5	-

Spearman’s nonparametric test. Correlation coefficients (r) are presented in the table.

Significant correlations are indicated by italics and * = *P* < 0.05 or ** = *P* < 0.01.

Tam = tamoxifen; 4OHtam = 4-hydroxytamoxifen; 4OHNDtam = 4-hydroxy-*N*-desmethyltamoxifen; NDtam = *N*-desmethyltamoxifen; NDDtam = *N*-desdimethyltamoxifen; TamNox = tamoxifen-*N*-oxide.

## Discussion

In rats with DMBA-induced breast cancer, tamoxifen treatment was associated with a significant increase in the expression levels of steroid receptors coactivators as well as the growth factor receptors HER-2 and HER-3. The upregulation of SRCs observed in the present study is in line with previous observations from a clinical trial on preoperative tamoxifen treatment in human breast cancer where tumors expressed significantly higher levels of especially SRC-3/AIB1, but also SRC-1 and SRC-2/TIF-2 mRNA compared to controls after 4 weeks of tamoxifen treatment [6]. In a clinical study on neoadjuvant treatment with aromatase inhibitors for 12-16 weeks, we have also found a significant increase of SRC-1 mRNA levels during endocrine treatment [18]. The observed effects of endocrine treatment on SRC expression in different model systems *in vivo* suggest that induction of coactivators is an early response to the blockage of ER mediated signaling in breast tissue. This concept is supported by data from *in vitro* experiments in which estrogen suppressed the mRNA and protein levels of SRC-3/AIB1 in MCF-7 cells by negatively regulating the transcription of SRC-3/AIB1, whereas 4OHtam increased SRC-3/AIB1 mRNA and protein level by inducing the transcription of the SRC-3/AIB1 gene and stabilizing the protein [24,25].

In the present study we also found an upregulation of HER-2 and -3 during tamoxifen treatment in DMBA-induced tumors which are sensitive to tamoxifen treatment. This is in line with the significant upregulation of HER-2 mRNA observed during aromatase inhibition in human breast cancer [18], although no significant difference in HER-2 mRNA expression was found in human breast cancer after neoadjuvant tamoxifen [6]. *In vitro* assays indicate that estrogen potentially downregulates HER-2 mRNA and protein expression [26-28] whereas estrogen deprivation could lead to increased HER-2 expression, possibly by competition between the ER and HER-2 enhancer for the same coactivator [29]. When SRC-1 is released from ER, the coactivator can instead facilitate transcription of HER-2 [29]. Conversely, the paired box 2 (PAX2) gene product has been shown to compete with SRC-3/AIB1 for the HER-2 enhancer. Silencing of PAX2 led to an increase in SRC-3/AIB1 bound to the HER-2 enhancer and significantly higher levels of HER-2 mRNA levels during tamoxifen treatment in breast cancer cell lines [30]. Higher mRNA levels of HER-1 and HER-2, but not HER-3, have been observed at the time of resistance in MCF-7 cells treated with tamoxifen for a prolonged period of time. Interestingly, the increase in mRNA levels could not be related to genetic amplification, but rather to changes in gene transcription [31].

The HER family members form homo- or heterodimers when activated, where the choice of dimerization partner in part is dictated by the ligand and the cellular levels of the different HER receptors. HER-2 is the preferred dimerization partner for the other HER members [32]. We found HER-2 mRNA levels to correlate with the mRNA levels of the other HER family members in endocrine sensitive breast cancer. Transgenic mice overexpressing HER-2 or mutant forms of HER-2 with capacity to malignant transformation of cells exhibit high levels of endogenous HER-1 or HER-3 compared to controls [33,34], suggesting a concomitant overexpression of the HER family members. Cooperation between the different HER family members has been shown to contribute to carcinogenesis, both *in vitro* and in human cancers, and they are co-expressed in several human malignancies, including breast cancer [20,34,35]. Interestingly, HER-2 and HER-3, which in this study were found upregulated during tamoxifen treatment, have been identified as a functional unit in experimental breast cancer models, where they cooperate to deregulate the G1 to S transition during cell cycle and thus stimulate mitosis [36]. Dimerization with HER-3 has been shown to be essential to the activation of HER-2 where downregulation of HER-3 inhibited the procarcinogenic effects of HER-2 by inactivating the PI-3 K/Akt pathway [37].

In line with the procarcinogenic effects of HER-2 and SRC-3/AIB1, clinical studies indicate that overexpression of HER-2 and SRC-1 or SRC-3/AIB1 is associated with tamoxifen resistance and reduced disease-free and overall survival [15,17,38]. A significant correlation between the mRNA levels of SRCs and HER-2 was observed, both in the present animal model and in previous clinical studies on endocrine responsive breast cancer [6,18]. It has been shown that the mitogen activated protein kinase (MAPK) dependent transcription factor Ets-2 downstream of HER-2, may regulate the transcription of HER-2 through interaction with SRC-1 [39]. We did not observe any significant change in the level of Ets-2 mRNA during two weeks of tamoxifen treatment. However, Ets-2 and SRC-3/AIB1 were correlated with each other and HER-4. Ets-2 and SRC-3/AIB1 have been found to be coexpressed in human breast cancer samples [38]. It has been shown *in vitro* that growth factors can upregulate the interaction between the coactivator SRC-1, SRC-3/AIB1 and Ets-2, leading to increased protein expression of HER-2 [38]. Hence, the increased expression of coactivators induced by tamoxifen treatment, as found in this and a previous clinical study [6], can through increased interaction with Ets-2 contribute to the induction of HER expression, as observed for HER-2 and HER-3 in this study. Work in cell lines has also demonstrated that overexpression of HER-2 in ER positive cells can result in resistance to tamoxifen [40] and that tamoxifen assumes estrogen agonistic properties in ER-positive breast cancer cells that express high levels of SRC-3/AIB1 and HER-2 [12]. The SRCs are recruited to the ER in presence of tamoxifen and an activated HER-2/MAPK system [41], which could lead to tamoxifen resistance [42,43]. Silencing of SRC-3/AIB1 with siRNA can significantly reduce the HER-2 stimulated cell growth, and restore tamoxifen sensitivity [44]. In the light of such data, interplay between the HER family receptors and SRCs represents a possible biological mechanism by which ER signaling may be preserved within cells during antiestrogenic treatment.

Observations of increasing SRCs mRNA levels in tumors sensitive to endocrine treatment, and association between high SRC levels and endocrine resistance may appear contradictory. However, induction of coactivator expression may represent an early response to endocrine therapy, whereas endocrine resistance normally develops over years. Changes in the intracellular environment and/or genetic instability could lead to constitutive activation of signaling pathways by which post-translational modifications of both ER and SRCs could affect molecular conformation, activation, intracellular localization and degradation. This would in turn influence the efficacy of tamoxifen. The activity of the tamoxifen-ER complex can be modulated by phosphorylation of ER and/or coactivators by kinases such as MAPKs found downstream of HER-2 [45]. Both SRC-1 and SRC-3/AIB1 are phosphorylated and transcriptionally activated by MAPKs that stimulate the recruitment of the cointegrator CBP/p300 and enhance the histone acetyltransferase activity of the SRCs *in vitro*[46,47]. It has been shown that phosphorylation is crucial for regulation of SRC-3/AIB1 mediated activity on steroid and growth factor signaling and malignant cell transformation [47-49].

Tamoxifen is a prodrug which is hydroxylated, demethylated and *N*-oxidated by the cytochrome P450 enzymes (CYPs) and flavin-containing monooxygenases in liver and other tissues. The hydroxylated metabolites 4OHtam and 4OHNDtam, the latter also known as endoxifen, have the strongest affinity for the ER [50,51] and are now considered to be tamoxifen’s main metabolites and effector derivatives [52,53]. However, tamoxifen metabolism varies substantially between species and strains [22]. Thus, as the effect of tamoxifen is dependent on its metabolism, it is important to characterize the tamoxifen metabolism in this animal model of tamoxifen treatment. The concentration of tamoxifen and some of its metabolites in tumor in this study are in line with previous studies in man and rats showing up to tenfolds higher concentrations in tissues [23]. Using LC-MS/MS technology we were now able also to measure tamNox. As opposed to the other metabolites, both NDDtam and tamNox were detected at lower concentrations than the parent drug in serum samples and tumor tissue. Interestingly, tamNox was the only metabolite with higher concentrations found in serum compared to tumor tissue. This may be explained by the *in vitro* observation that tamNox can easily be reduced back to tamoxifen in tissues [54]. This reduction of tamNox is catalyzed by numerous CYPs without major selectivity. In this animal model, 4OHNDtam was found at higher concentration than the other hydroxylated metabolites in both tumor and serum. Also in humans 4OHNDtam is the hydroxylated metabolite with the highest concentration in serum and tissues [23,55,56]. A limitation to the present study is the high concentration of tamoxifen and its metabolites observed compared to previous studies using rats [23,57,58]. The variability in drug and metabolite concentrations between studies can be explained by factors such as tamoxifen dose, duration of treatment and interstrain variability in uptake, deposition and metabolism of tamoxifen as related to the variability in expression and inducibility of CYPs during tamoxifen treatment [57]. However, it should be noted that the metabolite/parent drug ratios of NDtam and NDDtam and the accumulation of tamoxifen and metabolites in tumor tissue are in line with previous findings from clinical tamoxifen trials [59].

## Conclusions

We observed an induction of the SRCs, HER-2 and HER-3 expression during tamoxifen treatment in DMBA-induced, endocrine responsive breast cancer. There were significantly positive correlations between SRC-1, SRC-2/TIF-2 and HER-2, and between SRC-3/AIB1, HER-4 and Ets-2 mRNA levels in tumor tissue. Further, HER-2 mRNA was correlated with the gene expression of the other HERs, an observation which indicates the importance of studying all the HERs in breast cancer. DMBA-induced breast cancer may be a suitable model for studies on the cross-talk between HERs, ER and SRCs *in vivo*.

## Abbreviations

4OHNDtam, 4-hydroxy-N-desmethyltamoxifen; 4OHtam, 4-hydroxytamoxifen; AIB1, Amplified in breast cancer 1; CYP, Cytochrome P450 enzyme; DMBA, 7,12-dimethylbenz(a)anthracene; ER, Estrogen receptor; Ets-2, Erythroblastosis virus E26 oncogene homolog 2; HER, Human epidermal growth factor receptor; LC-MS/MS, Liquid chromatography-tandem mass spectrometry; MAPK, Mitogen activated protein kinase; NDDtam, N-desdimethyltamoxifen; NDtam, N-desmethyltamoxifen; RT-PCR, Reverse transcription-polymerase chain reaction; SRC, Steroid receptor coactivator; tamNox, Tamoxifen-N-oxide; TBP, TATA-box binding protein; TIF-2, Transcription intermediary factor-2..

## Competing interests

The authors declare that they have no competing interests.

## Authors’ contributions

LLHM participated in the collection of data in the animal model, the gene expression assays, statistical analyses, interpretation of data and drafted the manuscript. MHF participated in the gene expression assays and performed the protein measurements, assisted in interpretation of data and helped to draft the manuscript. JG performed the measurements of tamoxifen and metabolites. AM contributed to the gene expression assays and drafting of the manuscript. JG, THR and OAG participated in the design of the study and acquisition of data in the animal model. EAL and GM contributed to the conception and design of the study, analyses of data and critical revision of the manuscript before publication. All authors read and approved the final manuscript.

## Pre-publication history

The pre-publication history for this paper can be accessed here:

http://www.biomedcentral.com/1471-2407/12/247/prepub
